# Multi-Modality Treatment for Patients With Metastatic Gastric Cancer: A Real-World Study in China

**DOI:** 10.3389/fonc.2019.01155

**Published:** 2019-11-01

**Authors:** Lin Zhao, Jiarui Li, Chunmei Bai, Yongdu Nie, Guole Lin

**Affiliations:** ^1^Department of Medical Oncology, Peking Union Medical College Hospital, Chinese Academy of Medical Sciences and Peking Union Medical College, Beijing, China; ^2^Department of General Surgery, Peking Union Medical College Hospital, Chinese Academy of Medical Sciences and Peking Union Medical College, Beijing, China

**Keywords:** gastric cancer, multi-modality treatment, chemotherapy, gastrectomy, metastasectomy

## Abstract

**Introduction:** People with metastatic gastric cancer (GC) have a poor prognosis. The study aims to investigate the efficacy of multi-modality treatment for patients with metastatic GC.

**Methods:** We retrospectively identified 267 patients with stage IV gastric cancer who were treated with systemic chemotherapy: 114 received multi-modality treatments, 153 received systematic chemotherapy alone. The survival of these two groups was compared by log rank test, the independent prognostic factors were investigated using univariate and multivariate analyses.

**Results:** The median survival of metastatic GC patients who received multi-modality treatment was significantly longer than those who received systematic chemotherapy alone (18.4 vs. 11.4 months, *P* < 0.001). Multivariate analysis identified tumor histologic differentiation, CA19–9 level, previous curative resection, palliative gastrectomy, and metastasectomy as independent prognostic factors for overall survival. In the multimodality treatment group, patients who received palliative gastrectomy or metastasectomy had a longer survival than those who only received intraperitoneal chemotherapy or radiotherapy (21.6 vs. 15.2 months, *P* = 0.014).

**Conclusion:** Multi-modality treatments offer a survival benefit for patients with metastatic GC. Future prospective studies are needed to confirm the result.

## Introduction

Gastric cancer (GC) is the fifth most common cancers and the third leading cause of cancer death worldwide (1). Almost one million new cases of GC were diagnosed each year, and about 50% of them occurred in Eastern Asia (mainly in China) (2). Although an improvement of 5-years survival for GC was observed in the past 10 years, the prognosis of Chinese GC patients was still poor. Compared with a very high survival of GC in Korea (68·9%) and Japan (60·3%), the age-standardized 5-years relative survival was only 35.1% in China because most patients have inoperable disease at the time of initial presentation (3–5).

Gastrectomy is the only potentially curative therapy for resectable GC, but a major proportion of patients could have local or distant recurrence even after curative resection (6, 7). People with metastatic GC have a poor prognosis with a median survival time of around 4 months in the absence of systemic chemotherapy (5). For patients with metastatic diseases, it has been demonstrated in multiple trials and meta-analysis that systemic chemotherapy could extend overall survival (OS) by about 7 months more than best supportive care (8). Therefore, systemic chemotherapy is the standard treatment modality for stage IV GC patients. However, systemic chemotherapy still cannot provide significant survival benefits and the disease will progress ultimately. Although some clinical guidelines had recommendations about second- and further-line treatment regimen currently, there is still no global consensus across countries regarding the best therapeutic approach after failure of the first-line therapy (9, 10). The management of patients with metastatic GC is challenging.

Recent years, the number of options available for GC has been increasing rapidly (11). In addition to the development of new anticancer drugs, multi-modality treatments, such as palliative surgery, radiation therapy, intraperitoneal chemotherapy, and other approaches, are gaining support in the management of metastatic gastric cancer (12–18). However, despite these advances, their impact on long-term survival outcome for patients with metastatic GC remains unsatisfactory and the best form of multidisciplinary therapeutic strategy is still not established. In this real-world study, we will focus on the role of multi-modality treatment for patients with metastatic GC.

## Methods

### Study Design and Participants

Between December 2011 and November 2018, a total of 267 patients with initial stage IV gastric cancer in Peking Union Medical College Hospital were included consecutively. The eligibility criteria were: (1) histologically confirmed gastric or gastroesophageal junction (GEJ) adenocarcinoma; (2) distant metastases verified by enhanced computed tomography (CT) or other approaches; (3) over 18 years old; (4) ECOG 0-2; (5) received first-line systematic treatment. Patients were divided into two groups according to treatment modality: the multi-modality treatment group comprised 114 patients and the chemotherapy only group comprised 153 patients. The multi-modality treatment group was defined as patients who received both systematic chemotherapy and other modality treatments including palliative gastrectomy and metastasectomy, intraperitoneal chemotherapy, radiotherapy, radiofrequency ablation, and transarterial chemoembolization (TACE). The chemotherapy only group was defined as patients who received systematic chemotherapy alone. This study was approved by the ethics committee of Peking Union Medical College Hospital.

### Treatment

The treatment regimens of gastric cancer were mainly based on clinical guidelines of the Chinese Society of Clinical Oncology (CSCO) and the National Comprehensive Cancer Network (NCCN) (12, 19). Several cytotoxic agents are adopted to treat metastatic gastric cancer, including fluoropyrimidines (5-fluorouracil, S-1, capecitabine), platinum agents (cisplatin, oxaliplatin), taxanes (paclitaxel, docetaxel), and irinotecan. For some patients with human epidermal growth factor receptor 2 (HER2)-overexpressing tumors, trastuzumab is combined with cytotoxic chemotherapy. The method used by the hospital to test the HER2 status was immunohistochemistry and fluorescence *in situ* hybridization (FISH). Each patient's chemotherapy plan (including intraperitoneal perfusion) is individualized by senior medical oncologists in the department of medical oncology depending on the tolerance and response to different treatment regimens. All patients in this study received first-line chemotherapy. If patients had disease progression evaluated by medical oncologists and good performance status, they would consider receiving second- or further-line treatment. The palliative gastrectomy or metastasectomy were performed by surgeons from different specialties. Appropriate radiotherapy plan was determined by radiation oncologists based on the patient's general condition, irradiation field, possible normal tissue damage and so on. Radiofrequency ablation and TACE were performed by specialists from the department of radiology.

### Assessment and Follow-Up

The following assessment were applied every two to three cycles typically: detailed medical history, physical examination, serum tumor marker analysis, and contrast enhanced CT of the chest, abdomen and pelvis. Additional approaches such as positron emission tomography (PET) and bone scan were undertaken depending on a clinical suspicion of recurrence or metastasis. Radiographic tumor response is quantified by using Response Evaluation Criteria in Solid Tumors (RECIST).

All patients followed up every 3 months, either in a clinical visit or by telephone. At each the out-patient review, physical examination, necessary radiological examinations (enhanced CT or occasional PET-CT), and routine laboratory examinations were performed regularly. The follow-up data were updated until January 31, 2019.

### Statistical Analysis

All statistical analyses were performed using SPSS software (version 25, IBM Corp., Armonk, NY, USA). The OS is defined as the interval from the stage IV disease diagnosis to the latest follow-up or death. Continuous variables were assessed by *t*-test, and categorical variables were analyzed with Chi squared test. Related survival curves were constructed according to the Kaplan-Meier method, and a log-rank test was applied to compare these curves. The Cox proportional hazards regression model was adopted to identify the independent prognostic factors for survival, variables (*P* < 0.10) in univariate analysis were entered into multivariate analysis. A *P* < 0.05 was considered significant.

## Results

### Patient Characteristics

The baseline characteristics of patients at diagnosis of metastatic disease are shown in Table 1. The average age of included patients was 56.4 years old, and 67.8% of the participants were male. At the time of stage IV disease diagnosis, the metastatic sites included peritoneum (31.8%), liver (28.1%), Krukenberg tumor (14.2%), lung (6.0%), bone (9.4%), non-regional lymph nodes (43.8%), and other distant metastases (22.8%). The multimodality treatment group displayed a higher proportion of Krukenberg tumors (19.3% vs. 10.5%, *P* = 0.041) than the chemotherapy only group. Curative surgery was performed in 37.1% of patients before the diagnosis of metastatic disease. Neoadjuvant treatment and adjuvant treatment were given to 23.2 and 85.9% of patients who underwent curative resection separately. The median follow-up periods of multimodality treatment group and chemotherapy only group were 60.4 (95%CI: 50.4–76.5) months and 63.5 (95%CI: 44.7–82.3) months, respectively. There was no statistical difference between the multimodality treatment group and the chemotherapy only group in age, sex, histologic differentiation, HER2 status, tumor location, tumor marker level at diagnosis, number of metastatic sites, previous curative resection, and follow-up period.

**Table 1:** Baseline characteristics of patients with metastatic gastric cancer.

**Characteristic, *n* (%)**	**Total (*n* = 267)**	**Multimodality treatment (*n* = 114)**	**Chemotherapy only (*n* = 153)**	***P*-value**
Age (years), mean ± SD	56.4 ± 12.5	55.3 ± 11.9	57.1 ± 12.8	0.242
Sex				0.257
Male	181 (67.8)	73 (64.0)	108 (70.6)	
Female	86 (32.2)	41 (36.0)	45 (29.4)	
Differentiation				0.565
Well/median	61 (22.8)	28 (24.6)	33 (21.6)	
Poor	206 (77.2)	86 (75.4)	120 (78.4)	
HER2 status				0.520
Positive	54 (20.2)	26 (22.8)	28 (18.3)	
Negative	98 (36.7)	43 (37.7)	55 (35.9)	
Unknown	115 (43.1)	45 (39.5)	70 (45.8)	
Tumor location				0.496
Upper	79 (29.6)	29 (25.4)	50 (32.7)	
Middle	86 (32.2)	36 (31.6)	50 (32.7)	
Lower	94 (35.2)	45 (39.5)	49 (32.0)	
Diffuse	8 (3.0)	4 (3.5)	4 (2.6)	
CA19–9 level				0.184
Normal	161 (60.3)	74 (64.9)	87 (56.9)	
Elevated	106 (39.7)	40 (35.1)	66 (43.1)	
CEA level				0.062
Normal	144 (53.9)	69 (60.5)	75 (49.0)	
Elevated	123 (46.1)	45 (39.5)	78 (51.0)	
Metastatic site				
Peritoneum	85 (31.8)	43 (37.7)	42 (27.5)	0.075
Liver	75 (28.1)	27 (23.7)	48 (31.4)	0.167
Krukenberg	38 (14.2)	22 (19.3)	16 (10.5)	0.041
Lung	16 (6.0)	7 (6.1)	9 (5.9)	0.930
Bone	25 (9.4)	7 (6.1)	18 (11.8)	0.119
Non-regional lymph nodes	117 (43.8)	45 (39.5)	72 (47.1)	0.217
Other	61 (22.8)	25 (21.9)	36 (23.5)	0.925
Number of metastatic sites				0.529
1	138 (51.7)	63 (55.3)	75 (49.0)	
2	80 (30.0)	33 (28.9)	47 (30.7)	
≥3	49 (18.3)	18 (15.8)	31 (20.3)	
Curative surgery	99 (37.1)	46 (40.4)	53 (34.6)	0.339
Neoadjuvant treatment	23 (23.2)	10 (21.7)	13 (24.5)	0.257
Adjuvant treatment	85 (85.9)	39 (84.8)	46 (86.8)	0.225
Follow-up period (months), median (95%CI)	63.5 (50.4–76.5)	60.4 (48.3–72.5)	63.5 (44.7–82.3)	0.492

### Treatment

In the first-line systematic treatment, 4.1% of them received a single drug treatment (fluoropyrimidine, taxane, or irinotecan monotherapy), 78.3% of them received a two-drug combination (fluoropyrimidine, platinum, or taxane), and 7.5% of them received a three-drug combination (Table 2). Only 4.9% patients received trastuzumab targeted therapy. Second-line therapy was administered in about half of patients. Among the patients that received second-line chemotherapy, the most frequent regimen type was still two-drug combination (Table 2). Irinotecan or apatinib were prescribed in single agent or double agent combination regimen in second- or further-line treatment. The multimodality treatment group had a higher proportion of receiving third- (33.3 vs. 20.3%, *P* = 0.016) and further-line (13.2 vs. 5.9%, *P* = 0.040) systematic treatment than chemotherapy alone group. There was no statistical difference between these two groups in the chemotherapy regimen.

**Table 2:** Treatment regimens of patients with metastatic gastric cancer.

**Characteristic, *n* (%)**	**Total (*n* = 267)**	**Multimodality treatment (*n* = 114)**	**Chemotherapy only (*n* = 153)**	***P*-value**
First-line treatment	267 (100)	114 (100)	153 (100)	1.000
Single-agent (fluoropyrimidine or taxane)	11 (4.1)	3 (2.6)	8 (5.2)	
Double agent combination (fluoropyrimidine, platinum, or taxane)	209 (78.3)	89 (78.1)	120 (78.4)	
Taxane + platinum + Fluoropyrimidine	20 (7.5)	10 (8.8)	10 (6.5)	0.654
Trastuzumab involved	13 (4.9)	7 (6.1)	6 (3.9)	
Others	14 (5.2)	5 (4.4)	9 (5.9)	
Second-line treatment	139 (52.1)	67 (58.8)	72 (47.1)	0.058
Single agent (fluoropyrimidine, taxane, or irinotecan)	14 (10.1)	5 (7.5)	9 (12.5)	
Double agent combination (fluoropyrimidine, platinum, taxane, or irinotecan)	102 (73.4)	49 (73.1)	53 (73.6)	
Apatinib	12 (8.6)	7 (10.4)	5 (6.9)	0.683
Trastuzumab involved	8 (5.8)	5 (7.5)	3 (4.2)	
Others	3 (2.2)	1 (1.5)	2 (2.8)	
Third-line treatment	69 (25.8)	38 (33.3)	31 (20.3)	0.016
Single agent (fluoropyrimidine, taxane, or irinotecan)	13 (18.8)	5 (13.2)	8 (25.8)	
Double agent combination (fluoropyrimidine, platinum, taxane or irinotecan)	35 (50.7)	22 (57.9)	13 (41.9)	
Apatinib	13 (18.8)	5 (13.2)	8 (25.8)	0.255
Trastuzumab involved	7 (10.1)	5 (13.2)	2 (6.5)	
Others	1 (1.4)	1 (2.6)	0 (0)	
Further-line treatment	24 (9.0)	15 (13.2)	9 (5.9)	0.040

Among 114 patients who received multimodality treatment, 35 (30.7%) received palliative gastrectomy and 19 (16.7%) received metastasectomy (Table 3). The metastasectomy includes oophorectomy, adrenalectomy, hepatectomy, colectomy, and retroperitoneal lymphadenectomy. Fifty-two patients (45.6%) received palliative radiotherapy. In 37 patients who had peritoneal carcinomatosis and received intraperitoneal chemotherapy, fluoropyrimidine, or platinum agents were used most frequently. In addition, six patients with liver metastasis received TACE and six patients with liver metastasis received radiofrequency ablation.

**Table 3:** Treatment regimens of patients received multimodality treatment.

**Treatment regimens, *n* (%)**	**Multimodality treatment (*n* = 114)**
Palliative gastrectomy	35 (30.7)
Metastasectomy	19 (16.7)
Oophorectomy	15 (78.9)
Adrenalectomy	1 (5.3)
Hepatectomy	1 (5.3)
Colectomy	1 (5.3)
Retroperitoneal lymphadenectomy	1 (5.3)
Intraperitoneal chemotherapy	37 (32.5)
Platinum	18 (48.6)
Fluoropyrimidine	15 (40.5)
Taxane	4 (10.8)
Radiotherapy	52 (45.6)
Radiofrequency ablation	6 (5.3)
TACE	6 (5.3)
Others	2 (1.8)

### Survival

**Figure 1:**
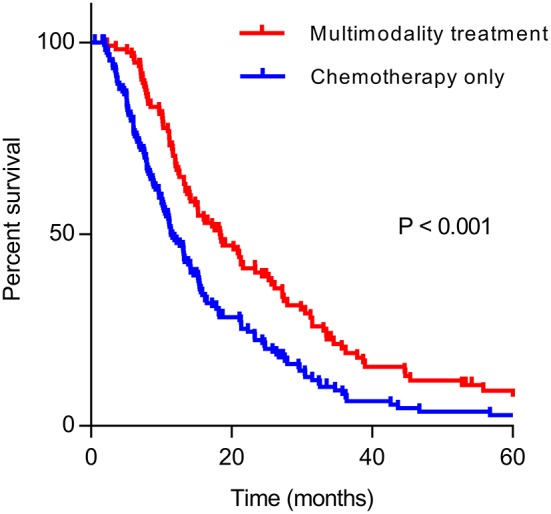
Kaplan–Meier curve of overall survival in multimodality treatment group and chemotherapy only group.

The median OS of patients who received multimodality treatment was prolonged significantly than patients who received systematic treatment only (18.4 vs. 11.4 months, *P* < 0.001, Figure 1).

**Figure 2:**
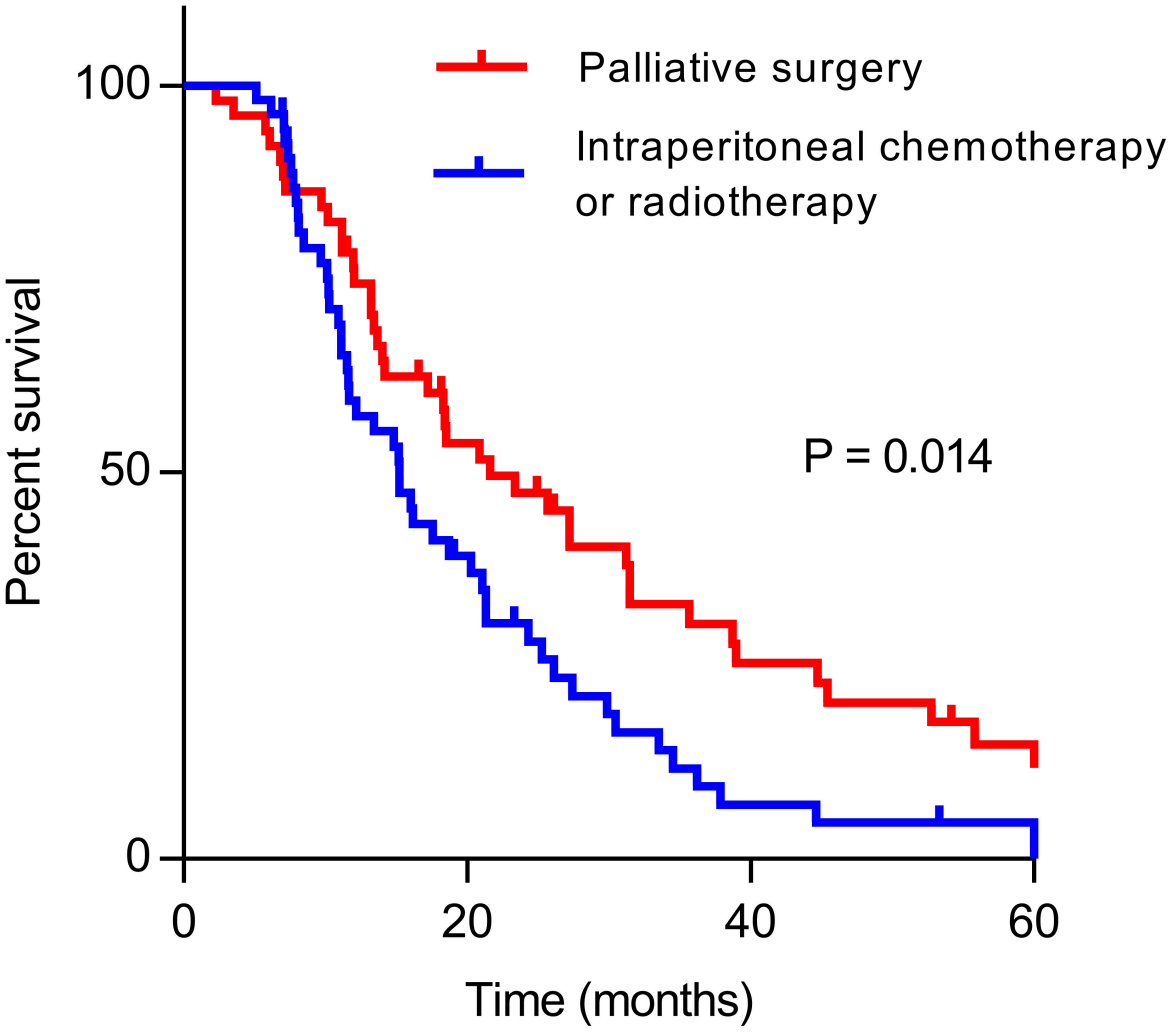
Kaplan–Meier curve of overall survival of patients who received palliative surgery (gastrectomy or metastatectomy) and other treatments (intraperitoneal chemotherapy or radiotherapy) in multimodality treatment group.

Univariate analysis of clinical prognostic factors that might influence the survival was performed on all included patients. The results demonstrated that factors such as differentiation, CA19–9 level, previous curative surgery, palliative gastrectomy, metastasectomy, and radiotherapy were correlated with OS (Table 4). Multivariate analysis was performed by incorporating related factors with Cox regression, and the results indicated that differentiation, CA19–9 level, previous curative surgery, palliative gastrectomy, and metastasectomy were the independent prognostic factors of OS. In the multimodality treatment group, patients who received palliative surgery (gastrectomy or metastasectomy) also had a longer survival than those who received intraperitoneal chemotherapy or radiotherapy (21.6 vs. 15.2 months, *P* = 0.014, Figure 2).

**Table 4:** Prognostic factors for OS of patients with metastatic gastric cancer on the univariate and multivariate analysis.

**Characteristic**	***n***	**MST (m)**	**Univariate analysis**		**Multivariate analysis**	
			**HR (95% CI)**	***P***	**HR (95% CI)**	***P***
Age	267	14.0	1.003 (0.993–1.014)	0.567		
Gender				0.865		
Male	181	13.4	Ref			
Female	86	15.2	1.024 (0.779–1.346)			
Location				0.305		
Upper	79	14.1	Ref			
Middle	86	13.1	1.260 (0.905–1.755)	0.171		
Lower	94	15.4	0.974 (0.702–1.353)	0.877		
Diffuse	8	14.2	1.423 (0.681–2.973)	0.348		
Differentiation				0.022		0.001
Well/median	61	21.3	Ref		Ref	
Poor	206	13.1	1.443 (1.053–1.977)		1.723 (1.231–2.410)	
CA19–9 level				<0.001		0.011
Normal	161	15.6	Ref		Ref	
Elevated	106	12.2	1.604 (1.219–2.110)		1.459 (1.089–1.956)	
CEA level				0.056		0.134
Normal	144	15.4	Ref		Ref	
Elevated	123	12.2	1.291 (0.993–1.678)		1.246 (0.935–1.660)	
Curative surgery				<0.001		<0.001
No	169	12.2	Ref		Ref	
Yes	98	18.3	0.605 (0.461–0.795)		0.588 (0.440–0.786)	
Second- and further-line chemotherapy				0.859		
No	128	11.3	Ref			
Yes	139	15.2	0.976 (0.751–1.270)			
Palliative gastrectomy				0.044		0.014
No	232	13.2	Ref		Ref	
Yes	35	18.4	0.661 (0.442–0.989)		0.590 (0.387–0.899)	
Metastasectomy				0.001		0.007
No	248	13.2	Ref		Ref	
Yes	19	35.6	0.423 (0.249–0.720)		0.468 (0.270–0.810)	
Intraperitoneal chemotherapy				0.474		
No	230	13.2	Ref			
Yes	37	17.6	0.872 (0.604–1.264)			
Radiotherapy				0.024		0.325
No	215	13.2	Ref		Ref	
Yes	52	17.6	0.682 (0.489–0.952)		0.842 (0.597–1.186)	

## Discussion

This real-world single center study showed that median survival of patients with stage IV gastric cancer who received multimodality treatment was significantly longer compared with those who received systematic therapy alone. In multivariate analysis, palliative gastrectomy, and metastasectomy were identified as independent improved survival factors, while second- and further-line chemotherapy, radiotherapy, and intraperitoneal chemotherapy were considered to be irrelevant.

Patients with stage IV GC usually have a poor prognosis and several randomized studies have provided evidence that first-line chemotherapy is more effective in terms of survival than best supportive care alone for patients with metastatic tumors (8). Therefore, patients with metastatic GC are primarily considered for systemic chemotherapy. However, treatment options after failure of standard first-line therapy are scarce and related benefit has to be weighed against treatment-related toxicities. Some randomized trials showed a survival advantage of the second- and further-line treatment over the best supportive care (20–23). However, such benefit was not seen in this real-world study even most patients still received two-drug combination regimen in the second- and further-line chemotherapy.

Surgery is not a standard treatment option for patients with stage IV GC, except for those who need alleviate symptoms such as bleeding and obstruction caused by the tumor (24). Although patients with metastases from gastric cancer are traditionally treated with systematic chemotherapy, this research and several retrospective studies indicated that gastrectomy or metastasectomy offered a more favorable survival compared with palliative chemotherapy alone by removing macroscopic lesions remaining (25–29). Even in the multimodality treatment group, patients who received surgery had a better survival than those who only received intraperitoneal chemotherapy or radiotherapy in our study. However, the clinical benefit of palliative surgery for stage IV GC is uncertain. A significant problem of these reports is selection bias. Candidates for surgical resection were more likely to have smaller disease burden and better performance status than those who received no surgical intervention. Recently, a phase III, randomized controlled trial (REGATTA trial) failed to show any survival benefit of gastrectomy in patients with advanced gastric cancer (30). Furthermore, patients undergoing gastrectomy had a significantly higher incidence of several serious adverse events related to chemotherapy in REGATTA trial. However, because of the presence of micrometastatic disease in advanced GC, it is more reasonable for advanced GC patients to receive the palliative surgery following a good response to systemic therapy. Palliative surgery in metastatic GC is a highly controversial topic, and the door to surgical resection are still not definitely closed (31). In the future, the effect of palliative resection in stage IV GC should be assessed as a component of multimodal treatment.

Peritoneal metastases are detected in about 30% of patients with advanced gastric cancer (32). Intraperitoneal chemotherapy is a reasonable strategy to approach peritoneal metastasis directly since it enables relatively high concentration of anticancer drugs to directly target cancer lesions in the peritoneum (33–35). In addition, patients with peritoneal metastasis can benefit from intraoperative chemotherapy administration combined with surgery (36). However, intraperitoneal chemotherapy in the current study yielded conflicting results and did not demonstrate a survival benefit. Similarly, the PHOENIX-GC trial failed to show statistical superiority of intraperitoneal paclitaxel in terms of overall survival (37). The possible clinical benefits of intraperitoneal chemotherapy for GC still need exploratory clinical trials.

In this research, palliative radiation therapy as a single modality in multivariate analysis also did not improve survival of metastatic GC patients. However, it is still attractive and has a well-defined role in symptomatic palliation in patients with unresectable gastric cancer, such as pain, bleeding, and obstruction (38). A population-based study demonstrated that radiation, surgery, or combination of both were associated with improved survival in advanced GC patients (39). The role of radiation therapy in stage IV GC remains controversial.

Our study has some limitations. First, this study was a retrospective design. Because of the retrospective nature, the selection bias exists inevitably and may influence the survival analysis. For example, patients with better status and less comorbidities are more likely to undergo more aggressive treatments, which may result in a better survival outcome. Second, this research was performed at a single institute. The indication for multi-modality therapy is various and dependents on the institute, the patients included in our center cannot represent the whole population of patients with stage IV GC who received multi-modality treatments. Third, as a real-world study, the heterogenous treatment schemes may be potential confounding variables that may influence the survival result although we have used the Cox regression analysis.

Up to now, it is impractical to cure stage IV GC, but the evidence is clear that using only one treatment modality cannot control this metastatic disease efficiently. Medical oncologists, surgeons, and radiologists from different disciplines should work together and offer the patients a comprehensive treatment plan to offer a chance of survival improvement. Optimal management of patients with metastatic GC is still challenging usually requires the integration of multidisciplinary therapeutic strategies either concurrently or sequentially.

## Conclusion

In conclusion, this real-world study provided the evidence that multi-modality treatment showed a significant survival benefit for patients with metastatic gastric cancer. Palliative gastrectomy and metastasectomy were independent prognostic factors for survival. In the future, large-scale prospective randomized clinical trials are needed to determine the optimal treatment strategy for stage IV gastric cancer.

## Data Availability Statement

The data used in this study are available from the corresponding author upon reasonable request.

## Ethics Statement

The studies involving human participants were reviewed and approved by Ethics Committee of Peking Union Medical College Hospital. Written informed consent for participation was not required for this study in accordance with the national legislation and the institutional requirements.

## Author Contributions

LZ, JL, CB, and GL conceived and designed the study. LZ, JL, and YN collected the data and wrote the manuscript. JL performed the statistical analyses. LZ, JL, and GL reviewed and revised the manuscript. All authors read and approved the final manuscript.

### Conflict of Interest

The authors declare that the research was conducted in the absence of any commercial or financial relationships that could be construed as a potential conflict of interest.
